# Using Psychophysiological Measures to Examine the Temporal Profile of Verbal Humor Elicitation

**DOI:** 10.1371/journal.pone.0135902

**Published:** 2015-09-02

**Authors:** Chris M. Fiacconi, Adrian M. Owen

**Affiliations:** The Brain and Mind Institute, Western University, London, Ontario, Canada; The University of Nottingham, UNITED KINGDOM

## Abstract

Despite its pervasiveness in popular culture, there remains much to be learned about the psychological and physiological processes that underlie our experience of humor. In the present study, we examined the temporal profile of verbal humor elicitation using psychophysiological measures of heart rate (HR) and facial electromyography (EMG). Consistent with recent prior research on cardiovascular changes to perceived humor, we found that HR acceleration was greater for jokes relative to non-jokes, and was positively related to the level of perceived humor elicited by these jokes. In addition, activity recorded from the *zygomaticus major* muscle that controls smiling was found to be greater for jokes relative to non-jokes. To link these physiological changes to the psychological processes that govern humor comprehension, we took the initial inflection point of the *zygomatic* EMG response as a marker for the onset of humor comprehension, and used this marker to probe the pattern of cardiovascular activity at this time-point. We estimated the onset of the humor response to occur during the initial HR deceleration phase, and found that jokes relative to non-jokes elicited a *decreased* HR response at this time-point. This result questions the previously forwarded notion that the psychological “moment of insight” that signals the start of the humor response is always associated with heightened cardiovascular activity. This discrepancy is discussed in relation to possible differences in the cognitive processes required to comprehend different forms of humor. At a broader level, our results also demonstrate the advantages of combining different psychophysiological measures to examine psychological phenomena, and illustrate how one such measure can constrain the interpretation of others.

## Introduction

Humor is a ubiquitous phenomenon in our daily lives. From TV shows, to radio commercials, to social interactions, there are few facets of life that are devoid of it. Humans are also remarkably adept at understanding humor–we can readily discern whether a particular joke was funny or not. Despite its pervasive presence and our extensive experience with it, however, there remains much to be learned about the psychological factors that govern our experience of humor. Most cognitively-oriented theoretical models of humor posit that joke comprehension involves three primary stages–the first being an initial *setup stage*, during which a particular meaning or theme is established, followed by a second *incongruity discovery stage*, during which the meaning-based expectancy created during the setup phase is violated, and finally, a *resolution stage*, during which this conflict in meaning is resolved through a re-interpretation process in which an alternate sense or meaning is derived [1,2,3]. This latter stage has been likened to a problem solving process, the end result of which is a “moment of insight” that corresponds to the time at which the intended meaning is realized, and marks the onset of the humor response.

Researchers have recently begun to shed light on the temporal dynamics of humor comprehension by employing psychophysiological measures of cardiovascular functioning to investigate when the critical moment of insight occurs [4]. In a recent study employing this approach, Lackner et al. [4] presented participants with humorous and non-humorous cartoons while simultaneously measuring transient cardiac responses to these stimuli. Participants were asked to indicate by button press the moment at which they were first able to comprehend the meaning of each cartoon. Critically, it was found that stimulus-locked changes in both heart rate (HR) and overall cardiac output were relatively greater for humorous cartoons relative to their non-humorous counterparts at 500 milliseconds *prior* to the time-point at which participants indicated their understanding of the cartoons. The authors interpreted this differential response as an autonomic marker of the “moment of insight” that begets humor comprehension, suggesting that measures of autonomic functioning may provide a promising avenue to examine the temporal coordination of the various processing stages involved in humor elicitation.

Transient changes in cardiovascular functioning have also provided insight into the physiological basis of the compelling affective experience with which humor is associated. Given that humor is typically conceptualized as a positive, approach-related emotion, it should result in the activation of the behavioural approach system [4,5], which is signalled by transient HR acceleration. Indeed, Lackner et al. [4] found that following an initial phase of HR deceleration reflecting stimulus-based orienting processes [6,7,8,9], there was greater subsequent HR acceleration for humorous cartoon stimuli, and that the extent of this humor-related cardiac acceleration was related to perceived humor. This finding is consistent with many other studies that have found greater subsequent HR acceleration when individuals are exposed to positively-valenced stimuli, including photos of loved ones [10,11,12] scenes [5,13,14] and sounds [15].

In addition to probing transient changes in cardiovascular responses, another psychophysiological measure that may complement measures of cardiovascular changes to humor is facial electromyography (EMG). Specifically, this technique involves recording activity of the *zygomaticus major* muscle that controls smiling [5,16,17,18]. This method provides a sensitive index not only of the extent of humor experienced in response to a joke, but also the time-course by which such humor becomes available, and has been used successfully in many other contexts to probe the presence of positive affect [19,20,21]. Importantly, activity of the *zygomaticus major* muscle can be conceptualized as providing an objective marker for the experience of humor that does not rely on self-reported feelings of amusement. This property of facial EMG makes it an ideal method with which to assess the temporal micro-genesis of the humor response and its cardiovascular concomitants.

The current study used facial EMG in conjunction with HR to examine the precise temporal profile of humor responses. In contrast to the majority of prior research on humor, we used verbal stimuli (jokes) rather than visual stimuli (e.g., cartoons, film clips), as verbal jokes unfold in a temporally structured and sequential manner that allows for precise control of when the joke meaning first becomes available. This property of verbal jokes renders them well suited to probe the time-course of humor processing and its corresponding autonomic signature. Recall that prior research investigating this issue with cartoon stimuli [4] used participants’ self-report of when they first “got” the joke as a marker for the onset of perceived humor, and compared the cardiovascular response to humorous and non-humorous cartoons 500 milliseconds (ms) prior to this timestamp to obtain a physiological index of the “moment of insight.” This approach assumes a) that participants’ self-reports are an accurate measure of the time at which humor is first gleaned (see Nisbett & Wilson [22], for an in-depth discussion of the accuracy of introspective reports) and b) that the time between the “moment of insight” and self-reported feelings of amusement is constant (i.e., 500 ms). To avoid these potential pitfalls, we sought to find a more accurate method for determining the critical “moment of insight” and its corresponding cardiovascular correlates by combining measures of cardiac functioning with measures of *zygomaticus major* muscle activity (facial EMG). To this end, we operationalized the “moment of insight” as the time corresponding to the initial inflection point of the *zygomatic* EMG signal post-punchline onset, as this time-point likely reflects the detection of the surprising shift in meaning associated with the punchline, and represents the onset of the ensuing humor response. We also recorded activity from the *corrugator supercilii* muscle that controls frowning, to gauge negative affect. This additional measure permitted us to ensure that the activity observed over the *zygomaticus major* muscle was in fact specific to positive affect, and not due simply to unrelated facial movements.

First, we predicted that jokes would elicit a greater humor response than non-jokes as evidenced by greater EMG activity over the *zygomaticus major* muscle. Second, we hypothesized that the humor response to verbal jokes would occur relatively quickly, as the gradually unfolding semantic context in the setup phase of the joke would facilitate detection of the critical incongruity in meaning that occurs upon onset of the punchline. Finally, we reasoned that the relative cardiovascular response to jokes and non-jokes at the time-point corresponding to the “moment of insight” may diverge from that reported by Lackner et al. [4] due to the potentially different time-course associated with verbal humor comprehension.

## Method

### Participants

Thirty-seven healthy undergraduate students from the University of Western Ontario participated in exchange for monetary compensation or course credit. There were 21 females, and participants’ mean age was 22.8 years.

### Ethics Statement

All experimental procedures were approved by the Research Ethics Board at Western University. All participants gave informed consent prior to their participation.

### Materials

Stimuli consisted of a total of 88 different sentences (see Appendix) with 44 belonging to each of two different categories: jokes, and non-jokes. These sentences were derived from those used in prior research that investigated the neural correlates of joke comprehension [23]. Both jokes and non-jokes were comprised of sentences that followed a common syntactic structure consisting of an initial setup line, followed by a punchline. The punchline was operationally defined in the current study as the critical phrase or word that allowed the global meaning of the sentence to be fully ascertained (see italicized words in the Appendix). Half of the joke stimuli and half of the non-joke stimuli were predicated on the meaning of an ambiguous word (e.g., Do you know what happens when frogs park illegally? They get *towed*) in the form of either a homonym, in which the same spelling can refer to different meanings (i.e., tank), or a homophone, in which the same pronunciation can refer to different meanings (i.e., mussel/muscle). The other half of each stimulus category did not contain any of these word types. Sentences in both categories were also designed to be closely matched in number of words, syllables, and syntactic structure. The duration of each sentence was on average 5.02 seconds, and ranged from 3.4 to 7.8 seconds. All sentences were recorded by a native English speaker using a lively prosody, rhythm, and intonation characteristic of jokes. Given that the primary purpose of the current study was to probe the psychological dynamics of humor comprehension using psychophysiological measures, we did not include these different types of joke and non-joke stimuli as a factor in our analyses.

Electrocardiography (ECG) signals were recorded using a BIOPAC MP150 system equipped with an ECG100C-MRI amplifier (BIOPAC Systems, Goleta, CA), and three Ag/AgCl electrodes. ECG electrodes were attached to participants using the standard three-electrode lead-II configuration in which electrodes were placed on the right collarbone, and the lower left and right ribcage. Facial EMG was recorded from the *zygomaticus major* and *corrugator supercilii* muscles, with a bipolar electrode setup consisting of two Ag/AgCl electrodes placed over each participants’ left cheek (*zygomaticus*), and left brow (*corrugator*). Electrode placement was based on published guidelines [24]. All signals were recorded using a BIOPAC MP150 system equipped with EMG100C-MRI amplifiers.

The experiment was conducted on a Dell desktop computer running E-prime 2.0 software, and stimuli were presented auditorily through a pair of noise-cancelling headphones.

### Experimental Procedures

Upon arrival at the laboratory, participants were first set up with the aforementioned psychophysiological measures. To facilitate acquisition of physiological signals, participants’ skin was gently abraded to remove excess skin and dirt prior to electrode placement. Before the experiment began, the experimenter explained in detail the instructions to each participant. Participants were asked to listen carefully to each sentence, and to then rate how funny they perceived each sentence on a scale from 1–7, with 1 being not funny at all, and 7 being extremely funny. The experimenter also emphasized that participants try and remain as still as possible so as to avoid introducing artifacts into the psychophysiological measures (i.e., deep breathing, fidgeting), but were nonetheless encouraged to react in a natural manner to each sentence. To allow participants to adjust to the experimental settings, they first listened to four practice sentences that were similar in structure to the remaining experimental sentences. Each sentence was followed by a brief silence (approximately 5 seconds) before the computer screen then prompted participants to indicate their funniness rating using the keys 1–7 on a keyboard. The rating screen was always presented 10 seconds after the onset of the sentence, thus leaving roughly 5 seconds of silence before each rating to minimize the impact of response-induced movement artifacts on psychophysiological recordings. To this end, participants were also asked to keep their fingers on the keyboard at all times so as to avoid large arm and hand movements. Following the rating, there was a 12–18 s inter-stimulus interval (ISI), chosen at random, prior to the onset of the next trial to facilitate the acquisition of the psychophysiological data. Each participant was presented with both sentence categories in a randomly intermixed order. The experiment lasted approximately 50 minutes, after which time participants were debriefed.

### Data Reduction and Analysis

The ECG signal was sampled at 2000 Hz, amplified, and band-pass filtered from 1 to 35 Hz. Estimates of HR in beats per minute (bpm) were derived online from the raw ECG signal by computing the time interval between each successive R-wave. For purposes of display and all analyses, HR data were averaged in 0.5-second segments for the 8 second interval following punchline onset, with each HR estimate weighted according to its precise duration [25]. To examine condition-specific changes in HR, data for each trial were baseline-corrected by subtracting the mean HR activity in the 2-seconds prior to the punchline onset from the mean value obtained for each 0.5-second segment that followed punchline onset. To assess the magnitude of HR deceleration, we measured the minimum baseline-corrected HR value within the first 4 seconds following punchline onset on a trial-by-trial basis. This 4-second window was chosen based on prior research examining emotion-related cardiovascular changes [5]. The acceleration phase of the HR response was calculated by subtracting the minimum HR value in the 0–4 second window from the maximum HR value- in the 4–8 second window following punchline onset. Eight participants were excluded from all analyses involving cardiovascular measures due to the presence of extensive movement artifacts in their ECG data.

The EMG data recorded from both muscle sites were sampled at 2000 Hz, amplified, and band-pass filtered from 30 to 500 Hz. The raw data were then rectified, and low-pass filtered at 2 Hz to obtain the envelope of the signal. We then baseline-corrected the resulting data by subtracting the mean EMG activity from the 1-s interval preceding the onset of the critical phrase/punchline from that occurring post-punchline to examine muscle activity specific to the processing of this critical information. To measure the magnitude of EMG activity in response to the punchline for each trial, we followed the approach taken by other researchers using facial EMG to probe changes in affective states (e.g., Bradley et al. [5]) by calculating the mean baseline-corrected muscle activity for the first 8-s post-punchline. The initial inflection point in the *zygomatic* response to the joke stimuli was defined as the time-point in the baseline-corrected EMG signal where the instantaneous slope first exceeded 2 μV/second within a 4-second window following punchline onset. This criterion was selected on the basis of visual inspection^2^, and was calculated for each participant based on their overall trial-averaged waveforms. Visual inspection consisted of plotting the first derivative of the baseline-corrected zygomatic EMG response together with the response itself to carefully select a criterion that corresponded to response onset as determined visually. This procedure was done for each participant to determine the optimal criterion that could be applied in the same manner to all participants. One participant was omitted from all analyses involving EMG activity due to the presence of many large artifacts in the EMG signal from both muscle sites, and eight others were excluded from the calculation of the mean initial inflection point because the instantaneous slope of their trial-averaged waveforms never exceeded 2 μV/second. These eight participants did not exhibit a noticeable smiling response to the joke stimuli.

## Results

### Behavioral

**Fig 1:**
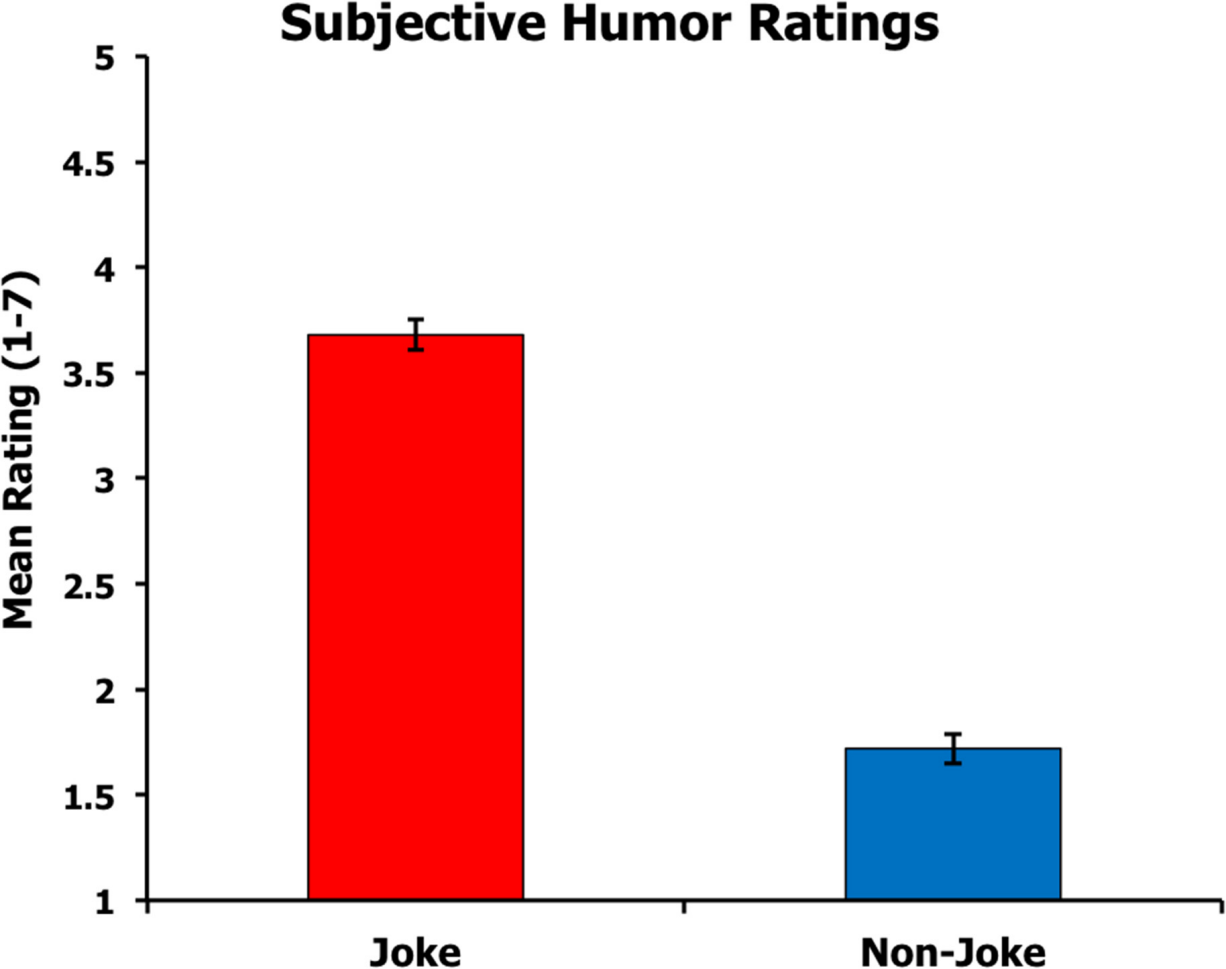
Mean humor ratings for each sentence category. Error bars represent standard error of the mean.

The mean humor ratings for each stimulus category are depicted in Fig 1. As expected, participants rated jokes to be funnier than non-jokes, *t*(36) = 13.8, *p* < .001.

### Facial EMG

**Fig 2:**
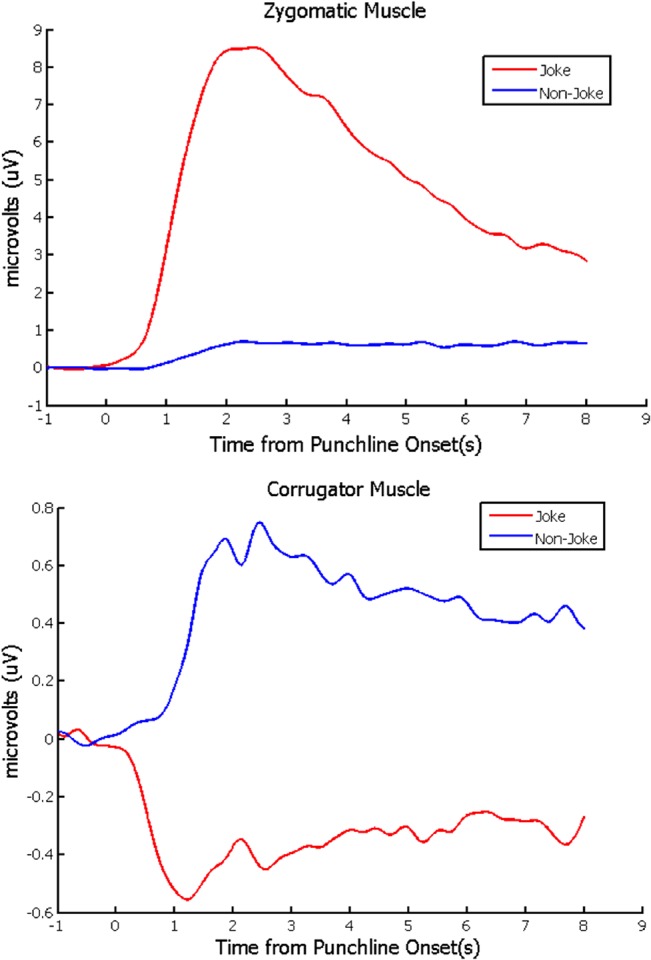
Grand-averaged baseline-corrected EMG activity time-locked to the onset of the punchline for zygomatic (top) and corrugator (bottom) muscle sites in response to each sentence category. Note the different scales used in each plot.

The mean EMG activity time-locked to the onset of the punchline for all sentences, for each muscle site, is depicted in Fig 2. We report the results for each muscle site separately.

For the *zygomaticus* muscle, jokes were associated with more activity than non-jokes, *t*(35) = 3.07, *p* = .002 (one-tailed)—a result consistent with the idea that jokes elicit a greater smiling response. The overall relationship between *zygomaticus* activity and subjectively perceived humor was assessed by computing for each participant the correlation between this activity and subjective humor ratings across all items. The mean correlation (.50) was significantly greater than zero, *t*(35) = 10.7, *p* < .001, indicating that the funnier a sentence was perceived to be, the greater a smiling response it elicited. To estimate the “moment of insight” in humor comprehension, we measured the latency of the initial inflection point of *zygomatic* activity following the punchline of joke stimuli based on each participant’s averaged waveform across all individual trials. As anticipated, the “moment of insight” was found to occur earlier (M = 803 ms, 95% CI [584, 1021]) than that reported in a previous study that estimated this time-point on the basis of participants’ self-report of the moment at which they first experienced amusement (~ 2.80 seconds [4]).

For *corrugator* EMG activity, non-jokes were found to elicit more activity than jokes, *t*(35) = 2.52, *p* = .017. With respect to the overall relationship between *corrugator* activity and subjectively perceived humor, the mean correlation across participants between *corrugator* activity and humor ratings (-.31) was significantly less than zero, *t*(35) = 6.75, *p* < .001, such that more humorous sentences were associated with less *corrugator* activity. These findings validate the EMG data collected from the *zygomaticus* muscle, suggesting that the observed activity in this muscle did indeed reflect smiling and not simply coincidental, extraneous muscle activity. Moreover, this result implies that in addition to increased positive affect, jokes may also be associated with less negative affect as compared to non-jokes.

### Heart Rate

**Fig 3:**
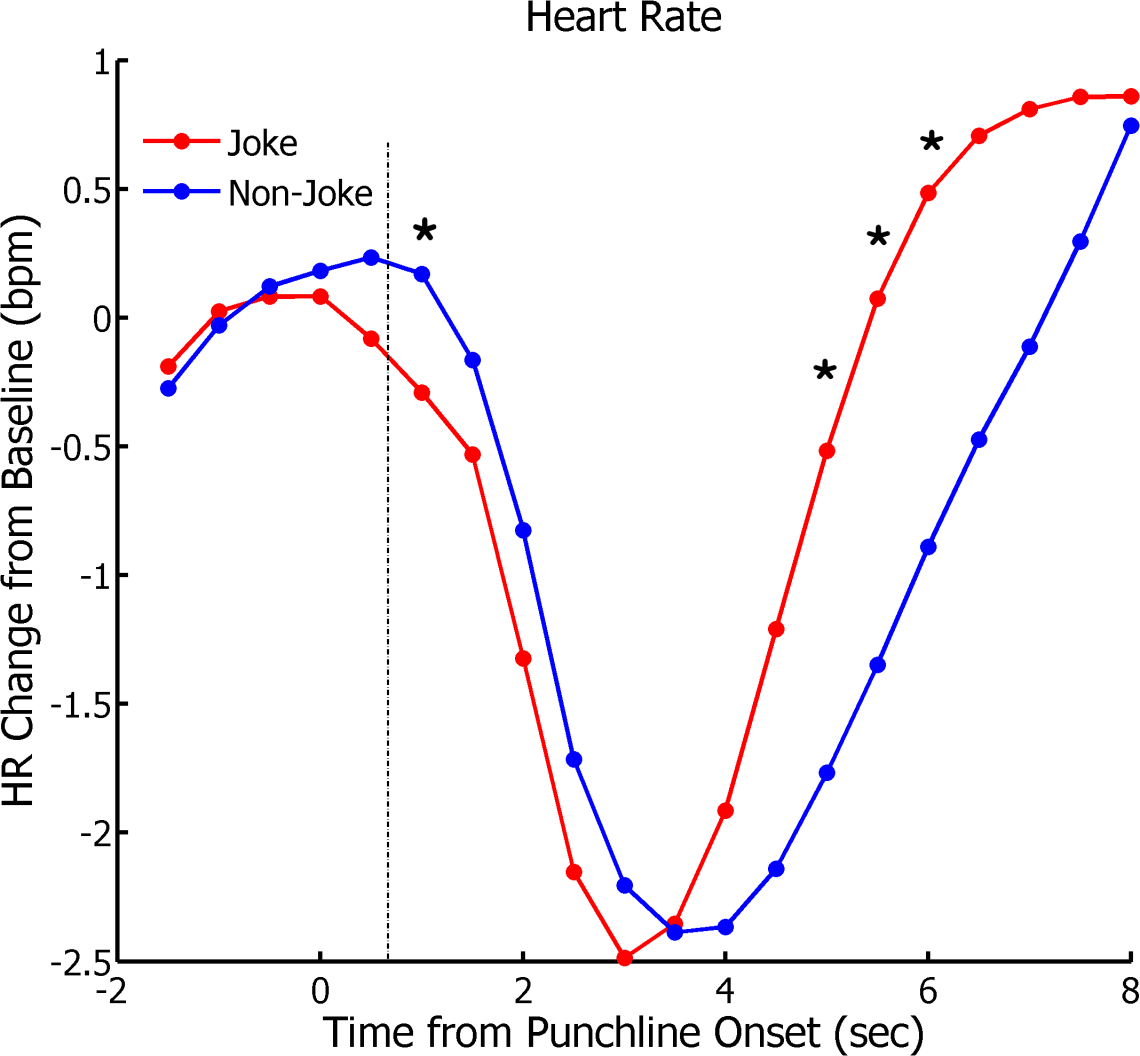
Grand-averaged baseline-corrected change in HR (bpm) time-locked to the onset of the punchline for each sentence category. The dotted line represents the mean response onset latency of the *zygomatic* EMG response. Asterisks represent Bonferroni-corrected significant differences between joke and non-joke stimuli at the corresponding time-points.

The mean HR change in beats per minute (bpm) time-locked to the onset of the punchline for all sentences, is depicted in Fig 3. To examine the time-course of the cardiovascular response to jokes and non-jokes, we first conducted a repeated-measures analysis of variance (ANOVA) including Condition (Joke/Non-Joke) and Time (16 half-second intervals post-punchline onset) as factors. This analysis revealed a significant main effect of Time, *F*(15, 420) = 27.95, *p* < .001, and a significant Condition x Time interaction, *F*(15, 420) = 11.54, *p* < .001, but no significant main effect for Condition (*p* = .15). To further probe the significant Condition x Time interaction, we conducted Bonferroni-corrected post-hoc t-tests to compare the effect of Condition at each time-point post-punchline onset. The HR response to joke stimuli was significantly slower than that to non-jokes at 1-second following punchline onset, *t*(28) = 3.56, *p* < .01, suggesting that HR deceleration is expedited for jokes. This result is particularly interesting in that this time-point corresponds closely to the onset latency of the initial inflection point of the *zygomatic* EMG response (803 ms), suggesting that the critical “moment of insight” is associated with a *decrease* rather than an increase in HR in our experimental paradigm. However, in line with Lackner et al. [4], we also found a significant increase in HR for jokes relative to non-jokes at the 5-second, *t*(28) = 3.35, *p* < .01, 5.5-second, *t*(28) = 3.55, *p* < .01, and 6-second time bins, *t*(28) = 3.28, *p* < .01, suggesting that jokes do indeed elicit greater HR acceleration. Critically, however, this increase in HR acceleration occurred well after the “moment of insight” as defined by participants’ *zygomatic* EMG response, and is therefore unlikely to represent this stage of cognitive processing. To supplement these analyses, we also calculated the magnitude of the HR deceleration and acceleration phases using the strategy employed by Bradley et al. ([5], see Data Reduction and Analysis section). Although the magnitude of HR deceleration did not differ between joke (-2.80 bpm) and non-joke stimuli (-2.61 bpm), *t*(28) = .67, *p* = .51, the latency of maximum deceleration for jokes (2.95 seconds) was significantly shorter than that for non-jokes (3.50 seconds), *t*(28) = 4.60, *p* < .001, suggesting that the start of the subsequent acceleration phase occurred earlier for jokes. The magnitude of HR acceleration was also larger for jokes (4.31 bpm) than for non-jokes (3.43 bpm), *t*(28) = 2.64, *p* = .013, consistent with the idea that jokes are associated with a more positive affective state, and greater activation of the behavioural approach system [4,5]. To assess the relationship between HR change and subjectively perceived humor, we correlated the magnitude of both the deceleration and acceleration phases and humor ratings for all trials on a trial-by-trial basis for each participant. For the initial deceleration phase, the extent of HR deceleration did not correlate with humor ratings, as the mean correlation (.026) did not differ from zero (*p* = .33). However, for the subsequent HR acceleration phase, the mean correlation between HR change and humor ratings across participants (.11) was significantly greater than zero, *t*(28) = 2.90, *p* = .008, suggesting that the cardiovascular changes in the acceleration phase in particular are linked to the degree of perceived humor.

### General Discussion

The current study sought to examine the temporal profile of verbal humor elicitation and its accompanying physiological changes by combining measures of cardiovascular functioning with facial EMG recordings of the *zygomatic* and *corrugator* muscles. The inclusion of facial EMG measures allowed us to constrain the interpretation of the transient cardiovascular responses associated with humor processing reported here and by others [4]. As expected, the HR response time-locked to punchline onset for both jokes and non-jokes was characterized by an initial deceleration phase that did not differ in magnitude across stimulus category, but was found to begin earlier, and was shorter in duration for jokes relative to non-jokes. Following this deceleration phase, the HR response to jokes was associated with greater acceleration relative to the non-jokes – a finding consistent with the idea that subsequent HR acceleration may index the degree of experienced positive affect. Further supporting this conclusion was the fact that the magnitude of HR acceleration, but not HR deceleration, was positively related to subjectively perceived humor. With respect to the facial EMG results, we found, unsurprisingly, that jokes elicited much greater *zygomatic* activity than non-jokes, with the opposite pattern holding true for *corrugator* activity. Most pertinent to the current study, we took the initial inflection point of *zygomatic* EMG activity as a marker for the critical “moment of insight”, which was found to occur 803 ms post-punchline onset on average. We then used this marker to compare the pattern of cardiovascular activity for jokes and non-jokes at this critical time-point. Unlike Lackner et al. [4], we found a decrease in HR for jokes as compared to non-jokes at this time-point, indicating that for verbal jokes, the “moment of insight” thought to mark the onset of humor comprehension is associated with cardiac deceleration.

A notable aspect of our results concerns the rapidity with which the humor response to verbal jokes was elicited. Using the inflection point of the *zygomatic* EMG activity as a marker of the “moment of insight,” we estimated that it took participants roughly 800 ms after hearing the punchline to “get” the joke. Interestingly, this estimated timeframe corresponds with that reported in a previous study that examined the neural dynamics associated with verbal humor comprehension using magnetoencephalography (MEG) [26]. Although jokes relative to non-jokes did not elicit a greater magnetic equivalent of the N400 (N400m), jokes were associated with a larger positive deflection occurring 700–1150 ms after the punchline that was localized to the right dorsolateral prefrontal cortex. Responses in this latter latency window have been linked to the detection and integration of semantic incongruities within the global sentence context [26,27], possibly reflecting a re-interpretive process whereby the incongruity is resolved with respect to the preceding context. Therefore, the greater positive activity within this window may reflect the critical incongruity resolution processes that result in the “moment of insight” in humor comprehension. Our *zygomatic* EMG data are consistent with this interpretation, suggesting that perceived humor is elicited rapidly following the presentation of the punchline.

The pattern of cardiovascular activity reported in the current study raises the question of how different phases of the transient cardiovascular response to jokes and non-jokes are related to the critical psychological processes that govern humor comprehension. To this end, the “moment of insight” as defined by the initial inflection point of the *zygomatic* EMG response provides an important constraint on the interpretation of the observed cardiovascular response. The finding that this inflection point occurred during the deceleration phase, prior to the point of maximal cardiac deceleration seems in contrast to the notion forwarded by Lackner et al. [4] that greater cardiac acceleration is associated with the “moment of insight” that begets humor comprehension. Indeed, a direct comparison of HR activity surrounding this critical time-point revealed a decreased HR for jokes relative to non-jokes. As noted earlier, cardiac deceleration has been linked to stimulus-based attentional orienting processes [6,7,8,9] that focus processing resources on salient events in the surrounding environment. Applied to the current findings, the initial HR deceleration we observed for jokes may reflect an orienting response to the unexpected, humorous meaning associated with comprehension of the punchline. By this view, the novelty of this shift in meaning captures attention, facilitating further processing and elaboration related to the contextual implications of the joke. The shorter duration of this deceleration phase for these stimuli may reflect the relatively greater opposing influence of positive affect which is known to be associated with cardiac acceleration [4,5,12], resulting in a faster shift from deceleration to acceleration. Indeed, we found that the extent of cardiac acceleration was positively related to the degree of perceived humor, suggesting that this phase tracks the positive affective experience associated with amusement. It is somewhat less clear as to the interpretation of the HR deceleration response to the non-jokes, as these stimuli do not contain an obvious shift in meaning. One possibility is that by presenting the jokes and non-jokes mixed together, participants formed an expectation that each sentence may contain a surprising punchline. In other words, participants were “waiting” for the humorous twist to occur at the end of each sentence. In the case of the non-jokes, this expectancy would be violated, which in turn may have triggered an orienting-like response. Regardless of the psychological interpretation of these different cardiovascular response phases, our findings clearly suggest that the “moment of insight” in verbal humor comprehension, as defined by the onset of the *zygomatic* EMG response, is associated with greater cardiac deceleration relative to non-joke stimuli.

Although we have emphasized some of the differences between our findings and those of Lackner et al. [4], it should be noted that some of our results are in fact consistent with this prior study. In particular, both studies found that following an initial HR deceleration phase for both stimulus categories, jokes elicited greater cardiac acceleration, and that the extent of this acceleration correlated with perceived humor. The primary point of contention across these two studies concerns how the pattern of cardiovascular response to humorous stimuli is related to the “moment of insight” that marks the moment at which the humor is apprehended. We suggest that the differences between the present set of results and those reported by Lackner et al. [4] likely arise from two factors. First, there are important differences in the stimulus materials utilized in each study. Recall that Lackner et al. [4] used humorous and non-humorous cartoon stimuli to examine the cardiovascular response to humor. Comprehending the intended humorous meaning depicted in these stimuli likely requires a greater degree of problem solving than the verbal stimuli used in the present study, as participants would need to discern the relevant perceptual attributes from the cartoon, integrate them with relevant schematic knowledge, and detect the critical semantic incongruity that elicits humor. In contrast, for the verbal joke stimuli, the relevant contextual information needed to ascertain the humor in the joke is provided for the participant, and unfolds gradually over time. Therefore, the detection and impact of the critical semantic incongruity introduced by the punchline is more immediate, as it can be rapidly resolved with respect to the preceding contextual information provided in the setup phase. Second, the cardiovascular responses reported by Lackner et al. [4] were measured relative to the onset of the stimulus, whereas in the current study they were measured relative to the onset of the punchline. Taking both of these considerations into account, it seems likely that the psychological processes reflected in the pattern of HR responses reported in the current study and that of Lackner et al. [4] would differ considerably, and as a consequence, the cardiovascular response associated with the “moment of insight” may also differ. We believe our findings suggest a more nuanced relationship between cardiovascular response patterns and cognitive processing in humor comprehension, such that a given response pattern (e.g., HR acceleration) may not always reflect the same psychological construct (e.g., “moment of insight”) across different stimulus contexts, and that understanding this relationship requires careful consideration of the information processing demands of the stimuli and task.

The results of the current study have important implications for our understanding of the physiological underpinnings of verbal humor comprehension. In particular, our findings suggest that the “moment of insight” associated with humor comprehension is not always associated with heightened cardiovascular activity, and may in fact be associated with HR deceleration depending on the nature of the humor-eliciting stimulus class and the requisite cognitive processes that are engaged. Therefore, when considered in isolation, the direction of HR change is not a reliable marker of the “moment of insight” in humor comprehension. At a broader level, the findings of the current study also illustrate the utility of using multiple psychophysiological measures to constrain our understanding of the cognitive and physiological processes that underlie humor comprehension, and to shed light on the relationship between these processes and the unique subjective experience with which humor is associated.

## Appendix

### Joke Stimuli

1 Do you know what happens when frogs park illegally? They get *towed*.
2 Do you know why cannibals don’t like clowns? Because they think they taste *funny*.
3 What did the teddy bear say when he was offered some dessert? No thank you, I’m *stuffed*!
4 Hey waiter, this coffee tastes like mud! Yes sir, it’s *fresh ground*.
5 What did the duck say when he’d finish shopping? Put it on my *bill*.
6 Why were the teacher’s eyes crossed? Because she couldn’t control her *pupils*.
7 Are you allowed to kiss a nun? Yes, but don’t get in to the *habit*.
8 Why do golfers wear two pairs of trousers? In case they get a *hole in one*!
9 How do we know the Native Americans were the first people in North America? Because they had *reservations*.
10 Did you hear about the guy who went to the seafood disco? He ended up pulling a *muscle*.
11 Doctor, I’ve got a strawberry stuck up my bum! No problem, I’ve got some *cream* for that.
12 Did you hear about the man that lost his whole left side? He’s *all right* now.
13 A guy walked in to a psychiatrist’s office, wearing only cling-film underpants. The psychiatrists said: well, I can *clearly see your nuts*!
14 Two aerials met on a roof, fell in love and got married. The ceremony was rubbish, but the *reception* was brilliant.
15 Did you hear about the young butcher who sat on a meant grinder? He got a little *behind* in his orders!
16 Two fish are in a *tank*. One turns to the other and says: do you know how to drive this thing?
17 Did you hear about the man who drowned in a bowl of muesli? He was pulled under by a strong current.
18 The ice cream man was found dead in his van, covered in chocolate sauce. The police say he had *topped* himself.
19 I said to the gym instructor: can you teach me to do the splits? How *flexible* are you? I said: I can’t make Tuesdays.
20 I saw a dermatologist about a nasty red patch on my skin. I asked him if it would get better; he said he didn’t want to make any *rash* promises.
21 Doctor, there’s a piece of lettuce sticking out of my bum! Is it serious? I’m sorry, but this is just the tip of the *iceberg*.
22 Did you hear the one about the giant that threw up? It’s *all over town*.
23 My mother told me she wanted grandchildren, I said: Mum, go for *it*!
22 How can you tell when a lawyer is lying? Easy! His lips *move*.
23 What are the worst three words you could hear while making love? Honey, I’m *home*!
24 There are three kinds of people: those who can count, and those who *can’t*.
25 Hey granny, what’s the best thing about being 104? No *peer pressure*.
26 What do you call an idiot hiding in a cupboard? The 1997 *hide-and-seek champion*!
27 Why did Cleopatra bathe in milk? She couldn’t find a cow tall enough for a *shower*!
28 Did you hear about the blind man that went bungee jumping? It scared the hell out of the *dog*!
29 Doctor, my eyesight is getting worse. It certainly is; this is the *post office*!
30 I want to die peacefully in my sleep like my grandfather; not screaming and yelling like his *passengers*.
31 Doctor to a patient: you are very sick! Can I get a second opinion? Yes, you’re *ugly too*!
32 Why did God create Man before Woman? Because he didn’t want any *advice*!
33 What is the definition of mixed emotions? When you see your mother-in-law backing off a cliff in your brand new *car*.
34 One cow says to another: are you worried about mad cow disease? The other says: why should I worry? I’m a *helicopter*!
35 Did you hear about the divorced Barbie doll they’re selling in shops? It comes with all of *Ken’s stuff*.
36 Doctor says: you’re in excellent health; you’ll live to be ninety. But doctor, I am ninety! Well, *that’s it*, *then*!
37 Teacher, would you punish me for something I didn’t do? No Sam. Well, that’s good; I didn’t do my *homework*.
38 My grandmother started walking 5 miles a day when she was 60. She’s 97 today; we haven’t got a clue *where she is*.
39 A sausage and egg are in a pan. The sausage says: Wow! It’s hot in here. The egg replies: Oh my God! *A talking sausage*.
40 Doctor, I keep thinking I’m a moth! You don’t need me; you need a psychiatrist. Yes I know; but I was just passing, and I saw your *light*!
41 A guy bought his wife a beautiful diamond ring. A friend said: didn’t she want a sports car? Yes, but where the hell was I going to find a *fake Porsche*?
42 Did you see the job advert for a telepath? It said: you will know where to *apply*.

### Non-Joke Stimuli

1. What happened to the letters in her book? They were put in alphabetical *order*.
2. What did you think of the club? It was better quality than the *golf balls*.
3. Why did the teacher buy the new foil? Because it would be easier to use than the other *sorts*.
4. Hey Mark, I hope the court is open today, I’d like to play *tennis*.
5. What did he say to the children about the tick? That it helped it *spread disease*.
6. What did the teacher think of the clip? She wanted to see the whole *movie*.
7. Doctor, good news! The corn has stopped growing. That’s perfect; now we can start the *harvest*.
8. What did the police say about the deed? That the businessman showed great courage during the *attack*.
9. Why did the employee struggle with the file? Because he had never worked with *metal before*.
10. What was the problem with the other coat? It was very difficult to put on with the *paint roller*.
11. Why was the guy annoyed about the cue? It was too short for the *Snooker table*.
12. What happened to the post? As usual, it was given to the best-qualified *applicant*.
13. The committee’s report was finally released; they concluded that the bars would be suitable for the local prison *windows and doors*.
14. What did the woman think when she first saw the coach? That it was unlikely he could teach the football team any new *skills*.
15. Did you hear the old man’s wish regarding the case? He hoped it would be remembered by the people on the *jury*.
16. When the newlyweds saw the seller of the cottage, they immediately thought they would not be able to trust *him completely*.
17. The tourist asked what she would see on her walk. She was told to look for a shell left over from the last *war*.
18. Doctor, do you see this protruding nail with a sharp edge? It’s used to tie back the *curtains in the window*.
19. There’s a lot of money to be won in the final game. I knew that an ace would be enough to win the *tennis match*.
20. I saw the manager leaving the office today. He said he hoped the mint he had chosen was suitable, and it would be able to produce the *coins in time*.
21. What did the chef do when he noticed the change in the kitchen? He collected it up and put it in his *piggy bank*.
22. Did the students continue the search? Yes, the passage they found had not yet been *translated*.
23. Why were the villagers angry? Because the local police force had been drastically *reduced*.
24. What did you think about the old church? That it should be kept *open*.
25. What did the chef think of the fresh herbs? He should have bought a much larger *amount*.
26. What was the salesman’s explanation? That the stains on the shirt had made the receipt *invalid*.
27. What was granny knitting? She was trying to finish the *scarf* for her grandson!
28. Why was the cage used to house a pet parrot? Because the *budgie had escaped*.
29. What did the gardener have to say? That the pond in the garden was *filthy*.
30. Why did the hiker struggle to find the lake? Because he had never used a *compass* before.
31. What did the tourists do in the sightseeing bus? They explored the old parts of the historic *town*.
32. Did you hear about last Friday’s storm? The tent was not enough to protect the *campus*.
33. Why was the man worried about the sail? Because it wouldn’t be large enough to *catch the wind*.
34. Did you hear about the farm? Following family tradition, it was inherited by the youngest *daughter*.
35. The young man thought about the blouse he had bought for his girlfriend. He concluded it would be the *perfect present* for her birthday.
36. What did she realize as she was walking across the field? That the path she had chosen was too muddy for *her sandals*.
37. Did you hear what mum said after the summer holiday was over? That a month had been too long to *spend together*.
38. As he walked to the old town center, he hoped the storm that was in the weather forecast would not get him *wet*.
39. Did you hear what the housewife saw from her window? She saw her three children playing with *new friends* from the neighborhood.
40. My girlfriend was listening to the radio, and heard a new song. It had a really catchy chorus and she decided to go out and *buy it*.
41. When the traveller walked out of the hotel, he saw the thick fog had descended again. He hoped it would lift quickly so that he could start his long *journey home*
42. The gambler counted up his remaining money. He realized that a win on the horses might be enough to repay all the *debts he owed*.
43. Doctor, did the results come back from my blood test? Yes, didn’t the nurse tell you? I’m sorry, but they confirmed the *diagnosis*.
44. Did the baker get his coffee? Yes, but it was far too cold for him to *enjoy*.

## References

[1] Suls J. A two stage model for the appreciation of jokes and cartoons In: Goldstein J, McGhee P, editors. Psychology of Humor. New York: Academic; 1972 pp. 81–100.

[2] Attardo S. The semantic foundations of cognitive theories of humor. Humor. 1997;10: 395–420.

[3] Vaid J, Hull R, Heredia R, Gerkens D, Martinez F. Getting a joke: the time course of meaning activation in verbal humor. J Pragmat. 2003;35: 1431–1449.

[4] Lackner HK, Weiss EM, Schulter G, Hinghofer-Szalkay H, Samson AC, Papousek I. I got it! Transient cardiovascular response to the perception of humor. Biol Psychol. 2013;93: 33–40. 10.1016/j.biopsycho.2013.01.014 23380334

[5] Bradley MM, Codispoti M, Cuthbert BN, Lang PJ. Emotion and motivation I: defensive and appetitive reactions in picture processing. Emotion (Washington, D.C.). 2001;1(3): 276–298. 12934687

[6] Libby WL, Lacey BC, Lacey JI. Pupillary and cardiac activity during visual attention. Psychophysiology. 1973;10(3): 270–294. 4702521 10.1111/j.1469-8986.1973.tb00526.x

[7] Graham FK, Clifton RK. Heart-rate change as a component of the orienting response. Psychol Bull. 1966;65(5): 305–320. 5325894 10.1037/h0023258

[8] Berntson GG, Boysen ST, Cacioppo JT. Cardiac orienting and defensive reactions: Potential origins in autonomic space In Campbell BA, Hayne H, Richardson R, editors. Attention and information processing in infants and adults: Perspectives from human and animal research. New York: Erlbaum; 1992 pp 163–200.

[9] Bradley MM. Natural selective attention: orienting and emotion. Psychophysiology. 2009;46(1): 1–11. 10.1111/j.1469-8986.2008.00702.x 18778317 PMC3645482

[10] Guerra P, Campagnoli RR, Vico C, Volchan E, Anllo-Vento L, Vila J. Filial versus romantic love: contributions from peripheral and central electrophysiology. Biol Psychol. 2011;88(2–3): 196–203. 10.1016/j.biopsycho.2011.08.002 21855602

[11] Guerra P, Vico C, Campagnoli R, Sánchez A, Anllo-Vento L, Vila J. Affective processing of loved familiar faces: integrating central and peripheral electrophysiological measures. Int J Psychophysiol. 2012;85(1): 79–87. 10.1016/j.ijpsycho.2011.06.004 21689694

[12] Vico C, Guerra P, Robles H, Vila J, Anllo-Vento L. Affective processing of loved faces: contributions from peripheral and central electrophysiology. Neuropsychologia. 2010;48(10): 2894–2902. 10.1016/j.neuropsychologia.2010.05.031 20678982

[13] Cuthbert BN, Schupp HT, Bradley MM, Birbaumer N, Lang PJ. Brain potentials in affective picture processing: covariation with autonomic arousal and affective report. Biol Psychol. 2000;52(2): 95–111. 10699350 10.1016/s0301-0511(99)00044-7

[14] Lang PJ, Greenwald MK, Bradley MM, Hamm AO. Looking at pictures: affective, facial, visceral, and behavioral reactions. Psychophysiology. 1993;30(3): 261–273. 8497555 10.1111/j.1469-8986.1993.tb03352.x

[15] Bradley MM, Lang PJ. Affective reactions to acoustic stimuli. Psychophysiology. 2000;37(2): 204–215. 10731770

[16] Iwase M, Ouchi Y, Okada H, Yokoyama C, Nobezawa S, Yoshikawa E, et al Neural substrates of human facial expression of pleasant emotion induced by comic films: a PET Study. NeuroImage. 2002;17(2): 758–768. 12377151

[17] Dimberg U, Thunberg M, Elmehed K. Unconscious facial reactions to emotional facial expressions. Psychol Sci. 2000;11(1): 86–89. 11228851 10.1111/1467-9280.00221

[18] Cacioppo JT, Petty RE, Losch ME, Kim HS. Electromyographic activity over facial muscle regions can differentiate the valence and intensity of affective reactions. J Pers Soc Psychol. 1986;50(2): 260–268. 3701577 10.1037//0022-3514.50.2.260

[19] Harmon-Jones E, Allen JJB. The role of affect in the mere exposure effect: evidence from psychophysiological and individual differences approaches. Pers Soc Psychol Bull. 2001;27: 889–898.

[20] De Vries M, Holland RW, Chenier T, Starr MJ, Winkielman P. Happiness cools the warm glow of familiarity: psychophysiological evidence that mood modulates the familiarity-affect link. Psychol Sci. 2010;21(3): 321–328. 10.1177/0956797609359878 20424063 PMC2948957

[21] Winkielman P, Cacioppo JT. Mind at ease puts a smile on the face: psychophysiological evidence that processing facilitation elicits positive affect. J Pers Soc Psychol. 2001;81(6): 989–1000. 11761320

[22] Nisbett RE, Wilson TD. Telling more than we can know: verbal reports on mental processes. Psychol Rev. 1977;84: 231–259.

[23] Bekinschtein TA, Davis MH, Rodd JM, Owen AM. Why Clowns Taste Funny: The Relationship between Humor and Semantic Ambiguity. J Neurosci. 2011;31(26): 9665–9671. 10.1523/JNEUROSCI.5058-10.2011 21715632 PMC6485420

[24] Fridlund AJ, Cacioppo JT. Guidelines for human electromyographic research. Psychophysiology. 1986;23(5): 567–589. 3809364 10.1111/j.1469-8986.1986.tb00676.x

[25] Graham FK. Constraints on measuring heart rate and period sequentially through real and cardiac time. Psychophysiology, 1978;15: 492–495. 693763 10.1111/j.1469-8986.1978.tb01422.x

[26] Marinkovic K, Baldwin S, Courtney MG, Witzel T, Dale AM, Halgren E. Right hemisphere has the last laugh: neural dynamics of joke appreciation. Cogn Affect Behav Neurosci. 2011;11(1): 113–130. Available: 10.3758/s13415-010-0017-7 21264646 PMC3047694

[27] Kuperberg GR. Neural mechanisms of language comprehension: challenges to syntax. Brain Res. 2007;1146: 23–49. Available: 10.1016/j.brainres.2006.12.063 17400197

